# Accuracy of Dementia Classification Algorithms in LMICs and HICs: A Systematic Review and Meta-analysis

**DOI:** 10.1093/geroni/igaf122.1614

**Published:** 2025-12-31

**Authors:** Wenjie Cai, Tsai-Chin Cho, Chihua Li, Kelvin Zhang, Sneha Mani, Lindsay Kobayashi, Alden Gross

**Affiliations:** Johns Hopkins Bloomberg University School of Public Health, Baltimore, Maryland, United States; University of Michigan School of Public Health, Ann Arbor, Michigan, United States; University of Michigan School of Public Health, Ann Arbor, Michigan, United States; Johns Hopkins Bloomberg University School of Public Health, Baltimore, Maryland, United States; University of Michigan School of Public Health, Ann Arbor, Michigan, United States; Johns Hopkins Bloomberg University School of Public Health, Baltimore, Maryland, United States

## Abstract

Compared with using clinical diagnosis information, dementia algorithms are a cost-effective alternative to classify dementia status. Numerous studies have developed dementia classification algorithms; however, many rely on specialized diagnostic information, such as MRI, PET, and blood/CSF biomarkers, that are costly and more available in high-income countries (HICs) compared with low- and middle-income countries (LMICs). In this systematic review and meta-analysis, we screened and synthesized the diagnostic accuracy of dementia classification algorithms in HIC and LMIC contexts based on demographics, health history, cognitive assessment, and/or informant reported cognitive and functional ability. We searched PubMed and PsycINFO for papers published between January 01, 2013 and February 28, 2025. After screening abstracts and full texts, 39 studies were included, of which 7 were conducted in LMICs. Fifteen studies reported sensitivity, specificity, and number of dementia cases, and thus were included in a meta-analysis: 53% (8/15) were community-based, and 80% (12/15) used machine learning algorithms. A hierarchical summary receiver operating characteristic (HSROC) model was used to construct summary ROC curves in LMICs (AUC=0.83) and HICs (AUC=0.95) separately. Dementia classification algorithms showed excellent performance in distinguishing dementia from normal cognition or mild cognitive impairment in both HICs and LMICs. The sparsity of published algorithms from LMICs highlights the need for further research on culturally and linguistically appropriate dementia classification algorithms in LMICs.

